# Cryo-SEM and confocal LSM studies of agar gel, nanoparticle hydrocolloid, mineral clays and saline solutions

**DOI:** 10.1038/s41598-022-14230-w

**Published:** 2022-06-15

**Authors:** Olena Ivashchenko

**Affiliations:** NanoBioMedical Centre, Adam Mickiewicz University, 61-614 Poznań, Poland

**Keywords:** Nanoscale materials, Structural materials, Techniques and instrumentation

## Abstract

Cryogenic electron microscopy became a powerful tool to study biological objects. For non-biological objects (solutions, gels, dispersions, clays), the polemic about interpretation of cryogenic microscopy results is still in progress splitting on two contradictive trends: considering structure as a near-real state of the sample or as freezing artefacts. In this study, a microstructure of a range of stable aqueous solutions and dispersions (agar, kaolin, montmorillonite, nanoparticles) was investigated by means of cryo-SEM and confocal LSM in order to compare cryo-fixed and unfrozen structures. Noticed correlation between these two methods for studied systems (agar, kaolin, montmorillonite, NPs) allowed to state that ordered microstructure is an inherent feature of these systems. Some inconsistencies in microstructure dimensions were discussed and prescribed to the differences in the bulk and interface layers. Supposedly, NaCl solutions also possess dynamic (femtosecond level) microstructure of neat water clusters and solvated Na^+^ and Cl^-^ ions that may have an impact on electrolyte abnormal properties.

## Introduction

Since discovering of cryogenic electron microscopy (cryo-EM) over thirty years ago, a lot of data has been accumulated in this field^1,2^. This imaging technique allows to observe specimens in micro- and nanoscale in their native aqueous environment, without drying that causes irreversible structure deformation. Observation becomes possible due to water solidification by cryogenic agents, i.e., cryo-fixation. The latter is a critical step in this technique: ultrafast freezing prevents ice crystal growth keeping the original structures intact. Cryo-fixation is usually achieved by plunging a specimen into cryo-agent (nitrogen slush, propane, ethane, etc.) which is followed by fracturing (to observe inner structure of the specimen) and sublimation (i.e., ice etching) that allows to observe structure without water layer. Today, cryo-EM technique became a powerful tool to study biological objects. A long-lasting discussion about congruence between their microstructure in cryo-fixed and non-frozen states was completed by consensus that cryo-EM results reflect near-real state of biological specimens in part of their denser structural elements (skeleton, membrane, etc.)^1,2^. The consensus was based on the accumulated data showing correlation between different imaging techniques. As for the liquid and gel-like biological components (mucus, inter- and extracellular liquids, etc.), the doubts still remain which may be prescribed to the difficulties in visualizing of low-density structures (low contrast, movability, etc.). One of the ways to solve this problem is to develop techniques that allow to observe wet samples in close-to-native conditions at high resolution. Toward this direction, the development of novel atmospheric SEM (ASEM), which is a combination of an inverted scanning electron microscope (with a detachable open-culture dish) and a fluorescence microscope, was reported^3^. The ASEM allows to quasi-simultaneously observe the same area from the top and bottom of the sample in aqueous solution (encapsulated in electron-permeable SiN film) at atmospheric pressure. Using this technique, neuronal connections, osteoblasts, endoplasmic reticulum, bone tissues, cell endocytosis and mitosis have been imaged^3,4^.

In comparison with biological objects, non-biological aqueous systems (e.g., solutions, dispersions, gels, clays) do not possess distinguished (dense) phase boundaries in water, and the interpretation of their cryo-EM results is still ambiguous and splitted into two contradictory trends. According to one trend, the microstructure observed at cryo-fixed samples is interpreted as a results of aqueous crystallites formation—as freezing artefact^5–11^. The latter appears when dissolved/dispersed substances diffuse towards ice crystal periphery leading to the formation of structure^7^. Within another trend, the microstructure of cryo-fixed samples is considered as near-real state. This point of view is often supported by correlation with optical or confocal laser scanning microscopy (LSM) images, as was shown, e.g., for foamed fracturing fluids, emulsions formed with apple sourced-pectin, nanoparticle dispersions, mineral clays^8,11–15^. Noticed differences in microstructure dimension (if compare LSM and cryo-EM results) were not interpreted and, thus, confusing^8^. The contradictory opinions of cryo-fixed microstructure indicate an inconsistency of experimental results with generally accepted concept for solutions and colloids as homogeneously distributed molecules/ions/particles in solvent^16^. Thus, the polemic about interpretation of cryogenic microscopy observations of non-biological samples is still in progress, and further research is needed to clear up this problem.

Another essential question is connected with water vitrification and/or the size of ice crystals formed during cryo-fixation. It is known that pure water can be vitrified as a thin layer or small droplet at fast freezing rates (also called “critical (minimal) cooling rates”), which is in the order of ~ 10^6^ K/s^2,17,18^. The presence of solutes reduces critical cooling rates by orders of magnitude. For a variety of solutes (ethanol, methanol, sodium chloride, glycerol, ethylene glycol, dextrose, etc.), the critical cooling rate is an exponential function of concentration and correlates with the solute’s Stokes radius^17^. In cryo-EM practise, however, cooling rates achieved are still far below theoretical limits^18^. It is believed that crystal ice volume fraction is small enough within the sample and does not prevent high-resolution reconstructions^2,18^. That is, for samples without distinguished interphase boundaries in water, doubts remain—what we really observe at cryo-EM measurements: sample structure or freezing artefacts? Do we observe dynamic solvate microstructure existed in liquid state which we “stopped” at the moment of cryo-fixation, or the microstructure was created at freezing moment? The problem is further complicated by obvious similarities between cryo-fixed structures of solutions and dispersions of different chemical nature of solutes (clay minerals, polysaccharides, colloidal gels, etc.): honeycomb, spongy and lamellae microstructures have been commonly observed^10,11,14,15^. These similarities in microstructure were usually interpreted as a proof for freezing artefacts existence which do not represent reality^8,9^.

In this study, the aim was to investigate a microstructure of stable aqueous solutions and dispersions of substances with different chemical origin (agar, kaolin, montmorillonite, ultrasmall iron oxide, and silver nanoparticles (MAg NPs)), by means of cryogenic scanning electron microscopy (cryo-SEM) and confocal LSM, and to compare cryo-fixed and unfrozen microstructures. In both methods, the samples were investigated as a drop with similar volume (not as a thin layer, which structure may differ significantly from bulk microstructure and depends on interphase interactions^19^). Further, cryo-SEM study of NaCl solutions (0.2–20 wt%) were performed, and highly ordered concentration-dependent microstructure was noticed. A supposition of a dynamic structuring of solvated Na^+^ and Cl^−^ ions and neat water clusters in liquid state was made.

## Results

**Figure 1:**
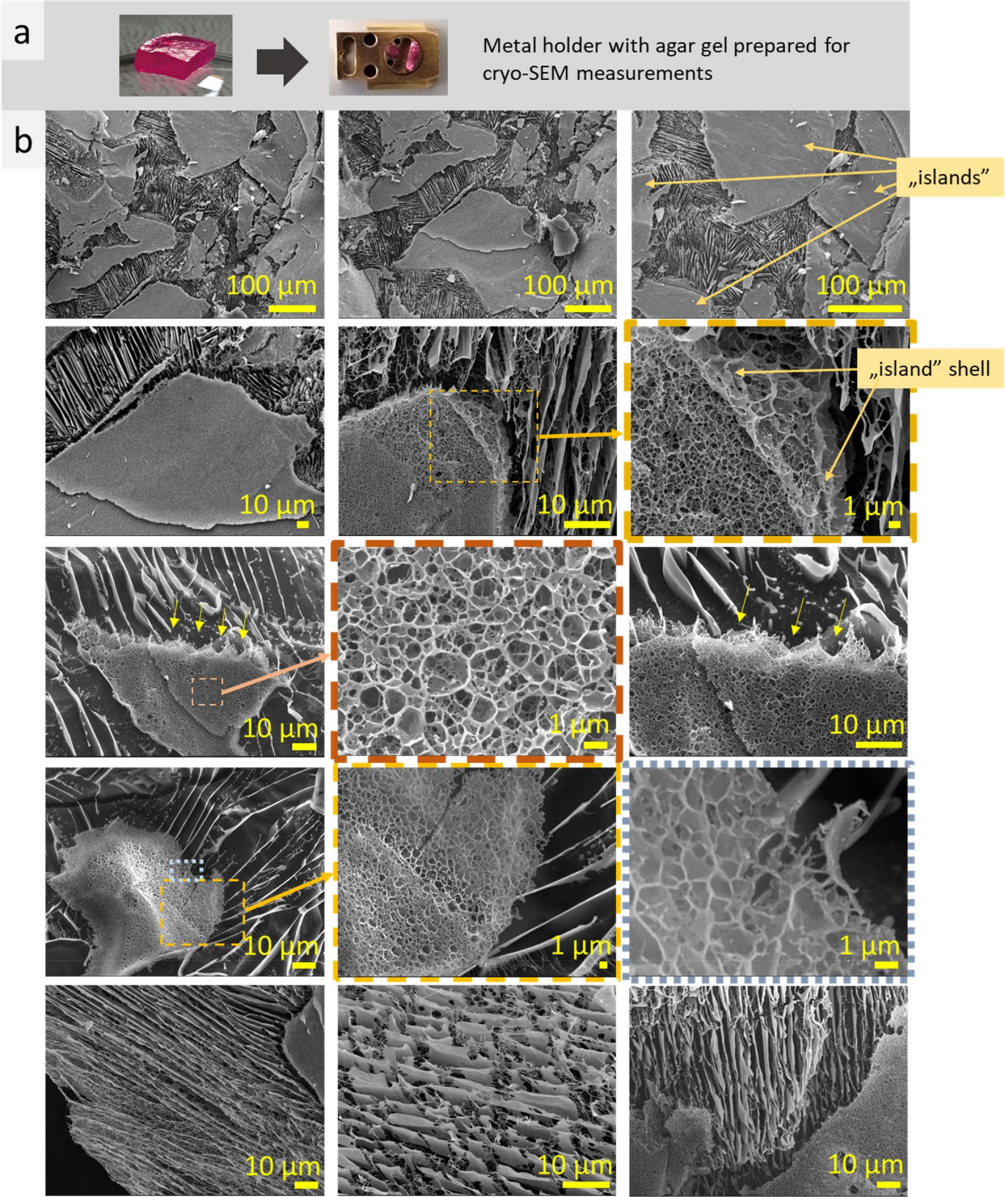
Photos of a piece of agar gel with rhodamine B and specimen shuttle with 10 mm aluminum holder with the gel prepared for cryo-SEM measurements (**a**); cryo-SEM images of agar gel with rhodamine B (**b**).

**Figure 2:**
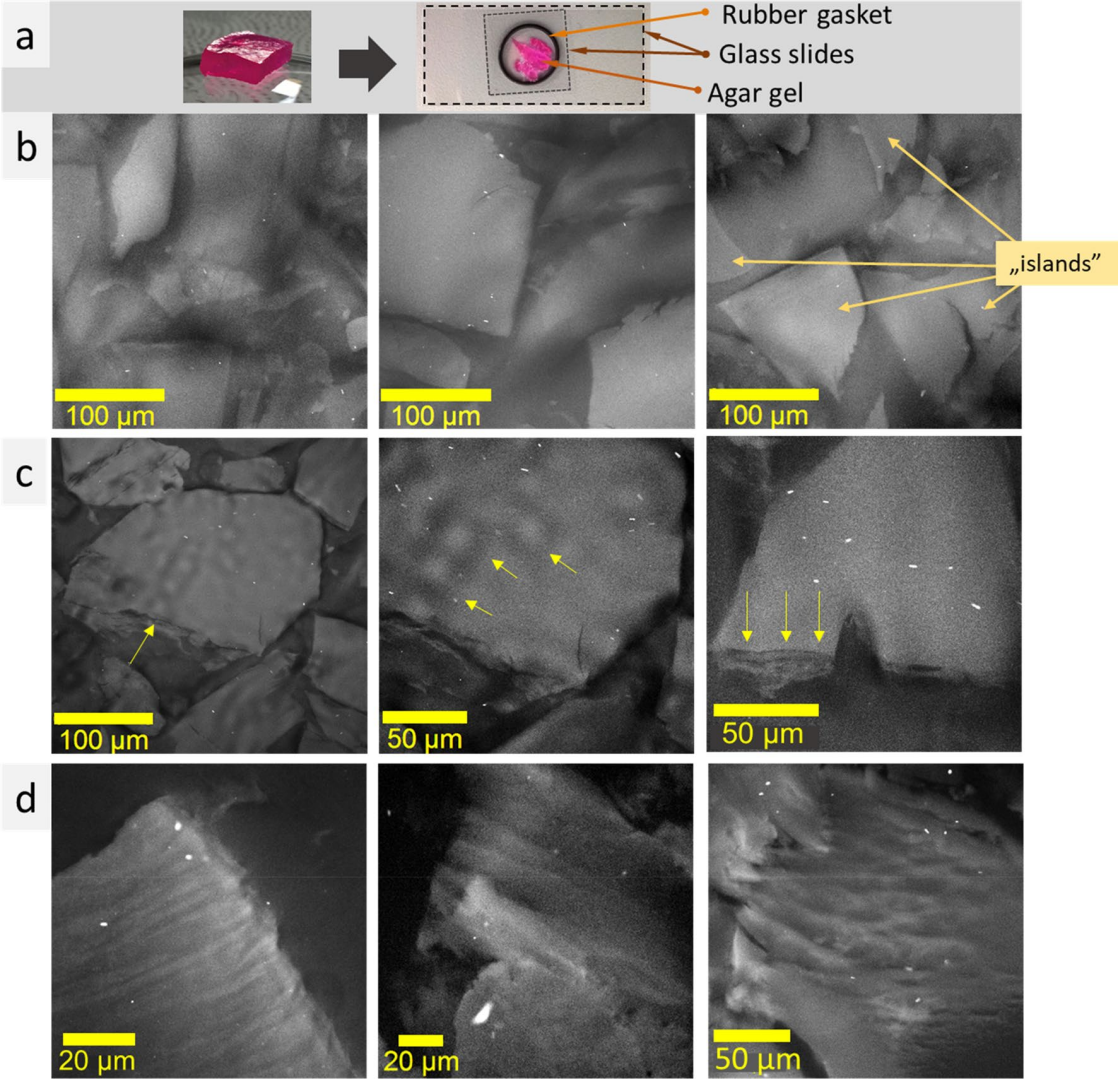
Photos of a piece of agar gel with rhodamine B and glass slides with the gel prepared for confocal LSM measurements (**a**); confocal LSM images of agar gel with rhodamine B (excitation wavelength 561 nm, laser power ~ 0.14 mW): “islands” (**b**), globular (**c** left and central images) and layered structures (**c** right); ordered microstructures (**d**). Contast and brigthness were adjusted.

In our study, specimens were kept voluminous during the cryo-SEM and confocal LSM measurements. This is important because the microstructure of thin layer differs significantly from that of bulk matters being strongly dependent on phase boundary interaction^19^. At cryo-SEM, a specimen was placed or poured into a hole in specimen stub (Fig. 1a). Sample preparation for LSM measurements was performed using rubber gasket to provide volume (increase layer thicknesses) (Fig. 2a). Fluorescent dyes (fluorescein, rhodamine B, Atto 550 NHS) introduced into the samples, allowed to use ultralow laser power (0.01–0.15 mW) that minimize sample heating, distortion and water evaporation during the measurements.

### Agar gel

Two types of microstructures, spongy and lamellar, that coincide like chaotically mixed “islands” with irregular shapes (20–400 µm in size) and jagged edges, were observed during cryo-SEM measurements of agar gel with rhodamine B (Fig. 1b). Spongy microstructure is finer, ~ 1 µm-scale, whereas lamellar is bigger, ~ 10 µm-scale. “Islands” are separated from bigger-scale lamellae microstructure by shell—a thin layer that differ from bulk microstructure of “island” (Fig. 1b, second row). Lamellae are strictly oriented at distances up to 100 µm, while the single lamellar elements are discontinuous (Fig. 1b, bottom row). Cryo-SEM measurements showed the presence of ordered microstructure in agar gel with rhodamine B.

Confocal LSM images of the agar gel with rhodamine B also revealed “islands” with irregular shapes (Fig. 2b). Similarly to cryo-SEM results, the “islands” are volumetric structures with irregular shapes 20–400 µm in size. In most cases, “islands” and inter- “island” space looked uniform and the microstructure ordering is not visible. Nevertheless, several distinct examples of microstructure ordering were found, mainly closely to the glass slide boundary, when the “island” lies on the glass or was “broken” (Fig. 2c–d). In particular, some of the “islands” were found to consist of globules (~ 20 µm in diameter) stacked in rows; the width of the row was ~ 40 µm (Fig. 2c, left, center). Such globules were not observed during cryo-SEM study. Also, layered structure was noticed on the “island” boundary; the distance between layers was of the order of 8–12 µm (Fig. 2c, right). Highly ordered elongated (with periodicity ~ 5 µm) and porous (~ 10 µm-scale) structures presented in Fig. 2d correlate with lamellae and spongy structures observed at cryo-SEM measurements (see Fig. 1b, bottom row). Thus, confocal LSM measurements of specimen, without any structure opening steps (water removing, fracturing, sublimation), provide the evidence of microstructure existence in unfrozen agar gel.

### MAg NPs hydrocolloid

**Figure 3:**
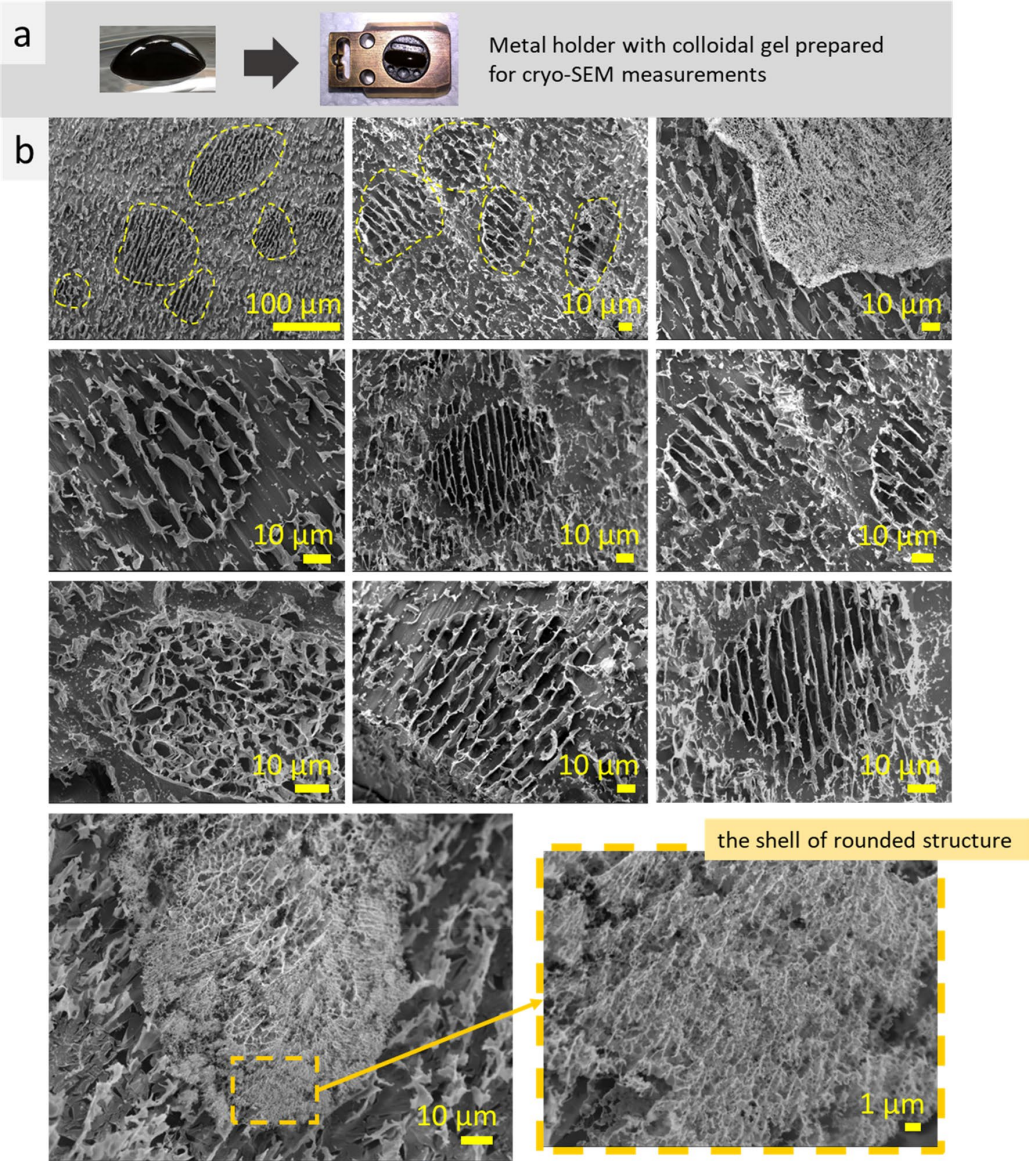
Photos of a drop of MAg NPs hydrocolloid with rhodamine B and specimen shuttle with 10 mm aluminum holder with the sample prepared for cryo-SEM measurements (**a**), cryo-SEM images of MAg NPs hydrocolloid with rhodamine B: rounded structures are circled by dotted lines (**b**).

In our previous studies, MAg NPs synthesized with natural extracts (Z. *officinale*, *A. muscaria*, *S. crispa*) have been shown to form stable hydrocolloids with distinctive microstructure^14,20,21^. Natural extract compounds (polysaccharides, proteoglycans, etc.) form surface layer on these NPs providing high stability of their hydrocolloids. The highly-ordered microstructure of the NPs hydrocolloids reveals mainly parallel stripes (lamellae) and sponge-like structures. Introducing of rhodamine B (for the sake of LSM imaging), as well as every change in hydrocolloid composition, may potentially influence hydrocolloid microstructure. That is why, cryo-SEM and LSM imaging was performed for the MAg NPs hydrocolloid with rhodamine B. Thus, cryo-SEM measurements revealed that MAg NPs with rhodamine B contain different types of microstructure, such as lamellae and sponge, with different scales (~ 1 µm and ~ 10 µm, between single elements) (Fig. S2). Cryo-SEM EDS measurements confirmed that the microstructure elements are formed by the NPs: Fe and Ag elements are in the microstructure elements, and O (from water) is between them (Fig. S2, a). High magnification images of microstructure element demonstrates that it consists of 100 nm-size globules (which are nanoparticles with solvation shell) (Fig. S2, b). Also, multiplicity of bigger rounded shapes, 30–100 µm in size, were observed (Fig. 3, marked by yellow dotted lines). These rounded structures consist of 10 µm-scale lamellae or spongy structures inside and fine-scale (≤ 1 µm) shell outside (Fig. 3, bottom images).

**Figure 4:**
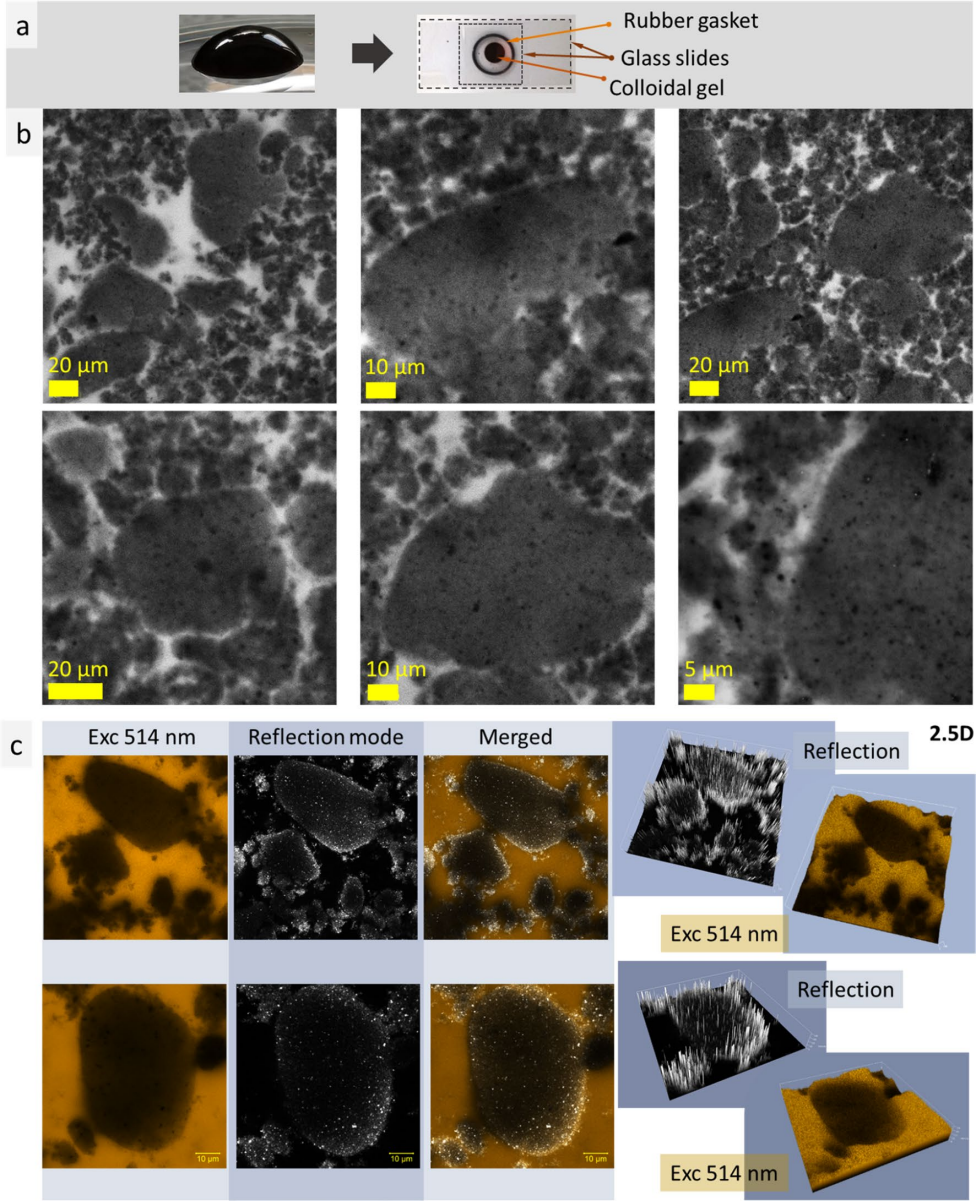
Photos of a drop of MAg NPs hydrocolloid with rhodamine B, and glass slides with rubber gasket with the specimen between them prepared for confocal LSM imaging (**a**); confocal LSM images (excitation wavelength 514 nm, laser power 0.01 mW) (**b**); double-channel measurements (excitation 514 nm and reflection mode), 2.5 dimensional (2.5D) simulation (axes *x*, *y*, intensity) (**c**).

Confocal LSM measurements of the MAg NPs hydrocolloid with rhodamine B revealed coexistence of two structures: irregular smaller-scale (~ 5–10 µm) and bigger rounded shapes (20–100 µm). Figure 4c presents confocal LSM images of this hydrocolloid at excitation wavelength 514 nm and in the reflection mode. Reflection mode showed clear edge contour of the roundish structure, 2.5 D simulation (x, y, intensity) confirmed intensive reflection on the edges of the observed structures. The observed bigger rounded shapes are similar in size and shape with that observed during cryo-SEM study. Uniformity of these roundish structures may be related to existence of tiny-structured shell that was noticed at cryo-SEM measurements (Fig. 4, bottom images).

### Kaolin clay

**Figure 5:**
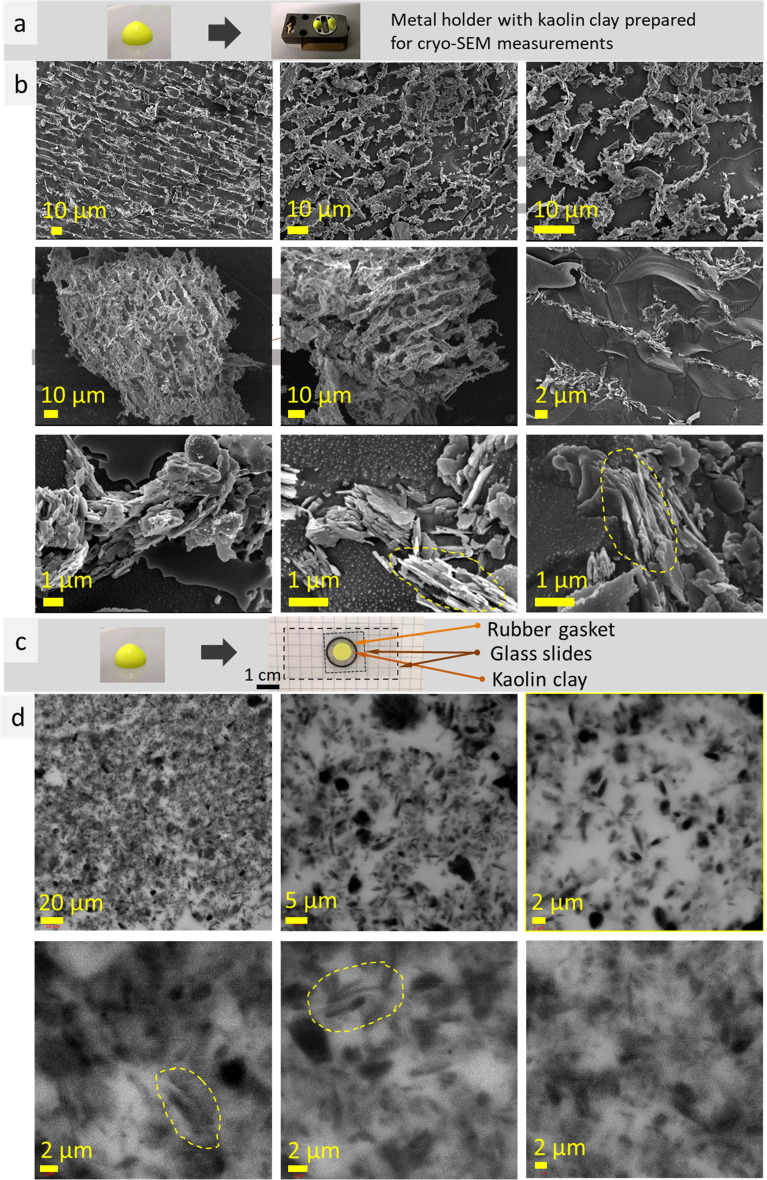
Photos of a drop of kaolin clay with fluorescein and specimen shuttle with kaolin clay in an aluminum holder prepared for nitrogen slush plunge-freezing (**a**), cryo-SEM images of kaolin clay with fluorescein (**b**). Photos of kaolin clay with fluorescein, glass slides with rubber gasket with kaolin clay between them for confocal LSM imaging (**c**); confocal LSM images obtained at excitation wavelength 405 nm, laser power 0.002 mW (light—kaolin, dark—water). Examples of parallel alignment of kaolin microplates are outlined in yellow dashed lines (**d**).

In the next set of experiments, kaolin clay with fluorescein was used for cryo-SEM and confocal LSM imaging. Kaolin (Al_2_(OH)_4_Si_2_O_5_) powder is hydrophilic, insoluble in water, and may form stable hydrocolloid—kaolin clay. To provide better fluorescence signal, two types of kaolin clays were prepared, with rhodamine B and fluorescein. Rhodamine B dye did not improve imaging (not shown) that may be related to poor rhodamine B adsorption on kaolin particles (this dye is well soluble in water, and exist mostly in water phase of kaolin clay (the supernatant is colored)). Fluorescein has better affinity to kaolin particles (colorless supernatant) and provides better contrast during confocal LSM imaging that may be related to its poor solubility in water (soluble in 1 M NaOH, 50 mg/ml) and better adsorption on kaolin particles. Cryo-SEM measurements of kaolin clay with fluorescein revealed lamellae and sponge microstructures (Fig. 5). The walls of these structures consist of flat micro plates—kaolin particles (circled with a yellow dotted lines in Fig. 5b).

Confocal LSM measurements revealed rather chaotic distribution of kaolin micro plates with characteristic feature—parallel alignment of kaolin micro plates (Fig. 5d, light contrast—kaolin, dark—water). Some examples of parallel alignment of kaolin micro plates are circled with a dotted line in Fig. 5d. The latter observation correlates with cryo-SEM measurements.

### Montmorillonite clay

**Figure 6:**
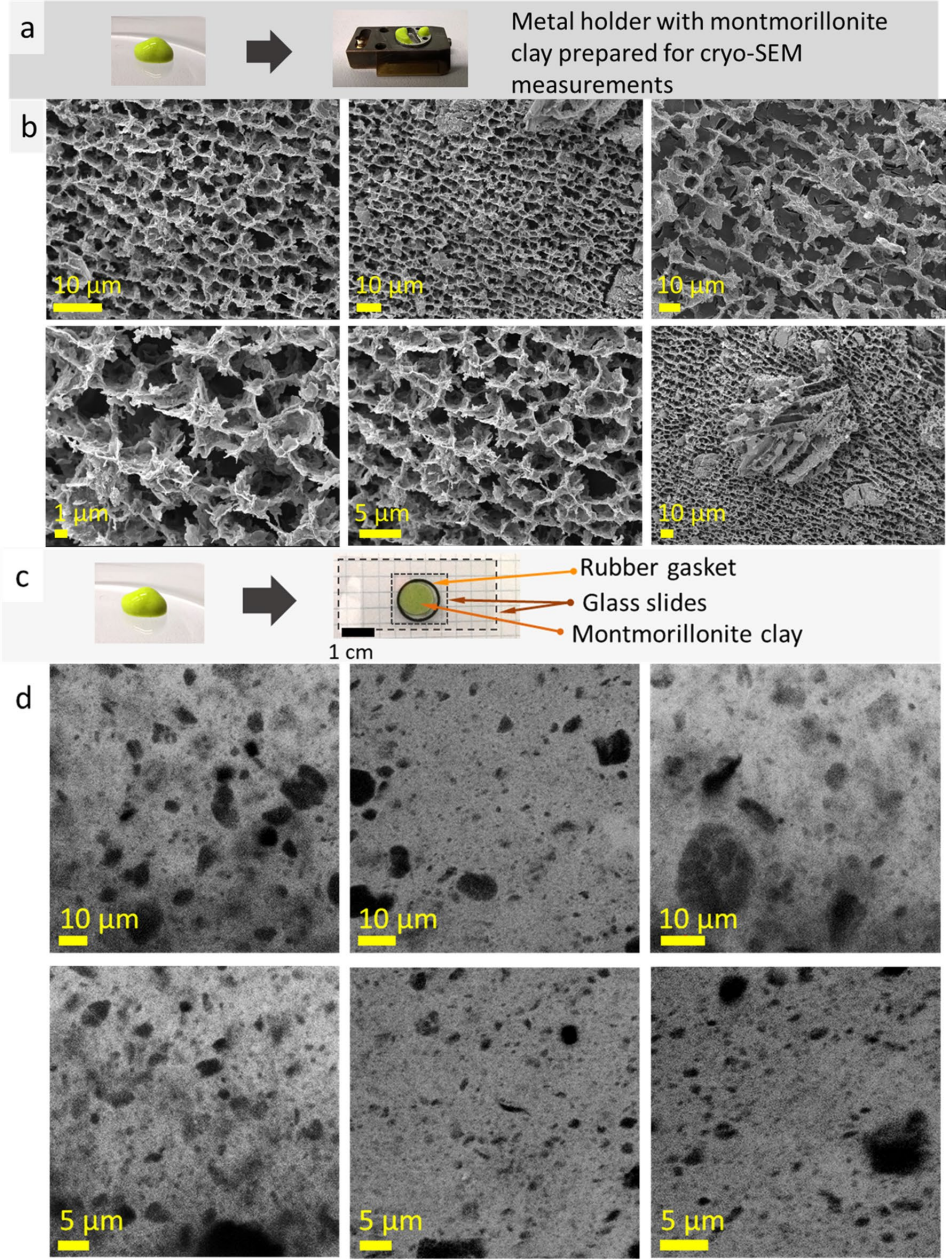
Photos of a montmorillonite clay with fluorescein, specimen shuttle with the sample for cryo-SEM (**a**), cryo-SEM images of montmorillonite clay with fluorescein (**b**). Photos of a montmorillonite clay with fluorescein, a drop between the glass slides with rubber gasket for confocal LSM imaging (**c**); confocal LSM images obtained at excitation 405 nm, laser power 0.002 mW (light—montmorillonite, dark—water) (**d**).

In continuation of the study, another mineral clay, montmorillonite was used for cryo-SEM and confocal LSM imaging (Fig. 6a,c). Montmorillonite ((Na,Ca)_0.33_(Al,Mg)_2_(Si_4_O_10_)(OH)_2_·*n*H_2_O) powder is hydrophilic, insoluble in water, and may form stable hydrocolloid—montmorillonite clay. To improve confocal LSM imaging, two samples of montmorillonite clay with fluorescent dyes, fluorescein and Atto 550 NHS ester, were prepared. Cryo-SEM measurements revealed porous spongy microstructure of montmorillonite clay (Fig. 6b). The scale of this microstructure differed in 3–4 times (Fig. 6b, first row, demonstrates ~ 4 µm- and ~ 11 µm-scale microstructures). Confocal LSM measurements (Fig. 6d) also revealed highly porous perforated structure that correlate with previously published results^2,8,9^. Herein, pore size was predominantly small, ~ 1 µm, with single inclusions of larger pores (5–20 µm).

**Figure 7:**
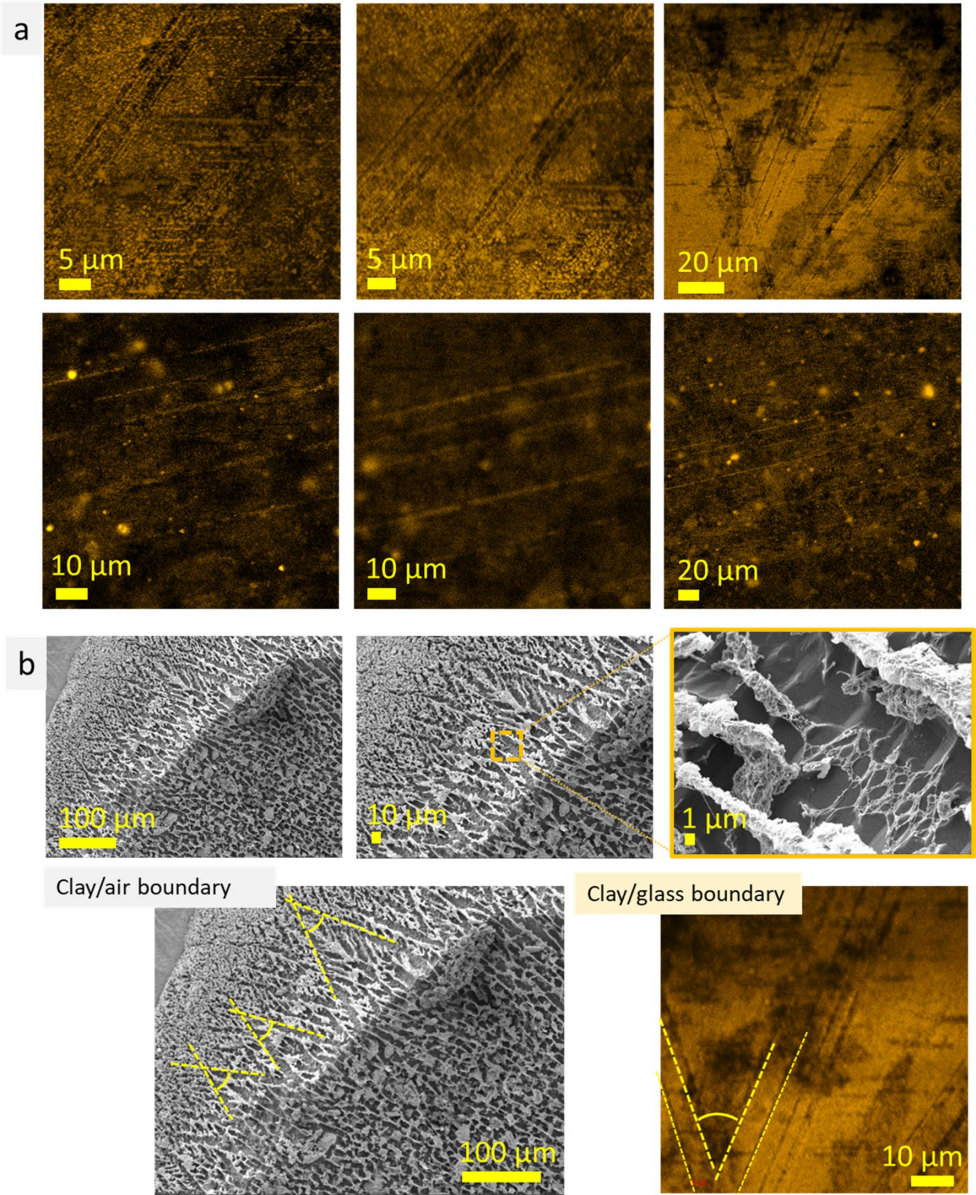
Confocal LSM images of montmorillonite clay with Atto 550 NHS ester (excitation wavelength 561 nm, laser power 0.012 mW, light—montmorillonite, dark—water) (**a**); cryo-SEM images of the surface (air/clay boundary) of montmorillonite clay, bottom images show similarity (acute angles marked by dotted lines) between structures observed by cryo-SEM (left) and confocal LSM (right) techniques (**b**) on montmorillonite clay/air and montmorillonite clay/glass boundaries.

Further, fluorescent dye Atto 550 NHS ester (MFCD05865403) was introduced into montmorillonite clay. This fluorescence dye is soluble in DMSO and has an affinity to montmorillonite particles (colorless supernatant). Visual observation of the montmorillonite clay with Atto 550 NHS ester revealed that addition of this dye decreased viscosity and increased fluidity. The latter was related to decrease of surface tension of montmorillonite clay due to addition of DMSO which surface tension is significantly lower than that of water (71.9 and 42.3 mN.m^−1^ (at 303.2 K) for water and DMSO, respectively)^22^. Due to the increased fluidity (fast particle movement), the confocal LSM measurements was performed without rubber gasket, covering the clay by glass cover that reduce thickness of the sample (~ 300 µm). The confocal LSM measurements (excitation wavelength 561 nm, ultralow laser power ~ 0.01 mW) revealed straight, long structures—lines (lamellae) (Fig. 7a). The lines were grouped together and highly parallel. They also formed acute angles (~ 38°). We relate the observation of lamellae structures to the improved interaction (affinity) between mineral clay and glass slide surface (due to the decreased surface tension and improved wettability of the clay). Interestingly, similar acute-angled structures were noticed on the montmorillonite clay surface, on the sample/air boundary, during cryo-SEM measurements (Fig. 7b). These results showed that montmorillonite clay may reveal different types of microstructure, namely, uniform porous perforated structure and highly parallel long lines (i.e., lamellae), depending on fluorescent dye added and, probably, thickness of the layer. These results show that the microstructure of montmorillonite clay observed during confocal LSM measurements strongly depends on interface interaction on the clay/glass boundary. To conclude, the results of cryo-SEM and confocal LSM measurements of montmorillonite clay with fluorescein correlate with each other, showing existence of well-organized microstructure.

### NaCl solutions

In continuation of the study, ultrapure water and NaCl solutions (0.2–20 wt%) was investigated using cryo-SEM technique. Ultrapure water revealed homogenous dotted surface (Fig. S3, first row), whereas NaCl solution (0.2 wt%) revealed discontinuous lamellar structures with thin walls; distance between lamellae was 10 ± 2 µm. At NaCl concentration 0.8 and 0.9 wt%, the lamellae walls became thicker and more continuous. Lamellae structures formed domains which are oriented at different angles to each other. When NaCl concentrations was in the range from 1.1 to 1.5 wt%, the bridges between lamellae appeared which transformed lamellar structure to cell-like. Herein, the wall thickness increased and a plurality of holes in the walls was still observed. At NaCl concentrations 2.0 and 5.0 wt%, the microstructure became completely cell-like. The cell-like elements are elongated, their transverse dimension is approximately 10 ± 2 µm. At NaCl concentrations 10.0 and 20.0 wt%, the size of cell-like elements decreased (transverse dimension 5‒8 µm) and the wall thickness further increased. Longitudinal and transverse dimensions of elemental cells approached each other (Fig. S3). Such a feature as microholes in the walls was noticed for NaCl solutions in concentration range 0.8–2.0 wt% (Fig. S4). The presence of microholes (0.5–2.0 µm in diameter) may be prescribed to air bubbles or considered as a specific feature that provide continuity of the microstructure walls.

**Figure 8:**
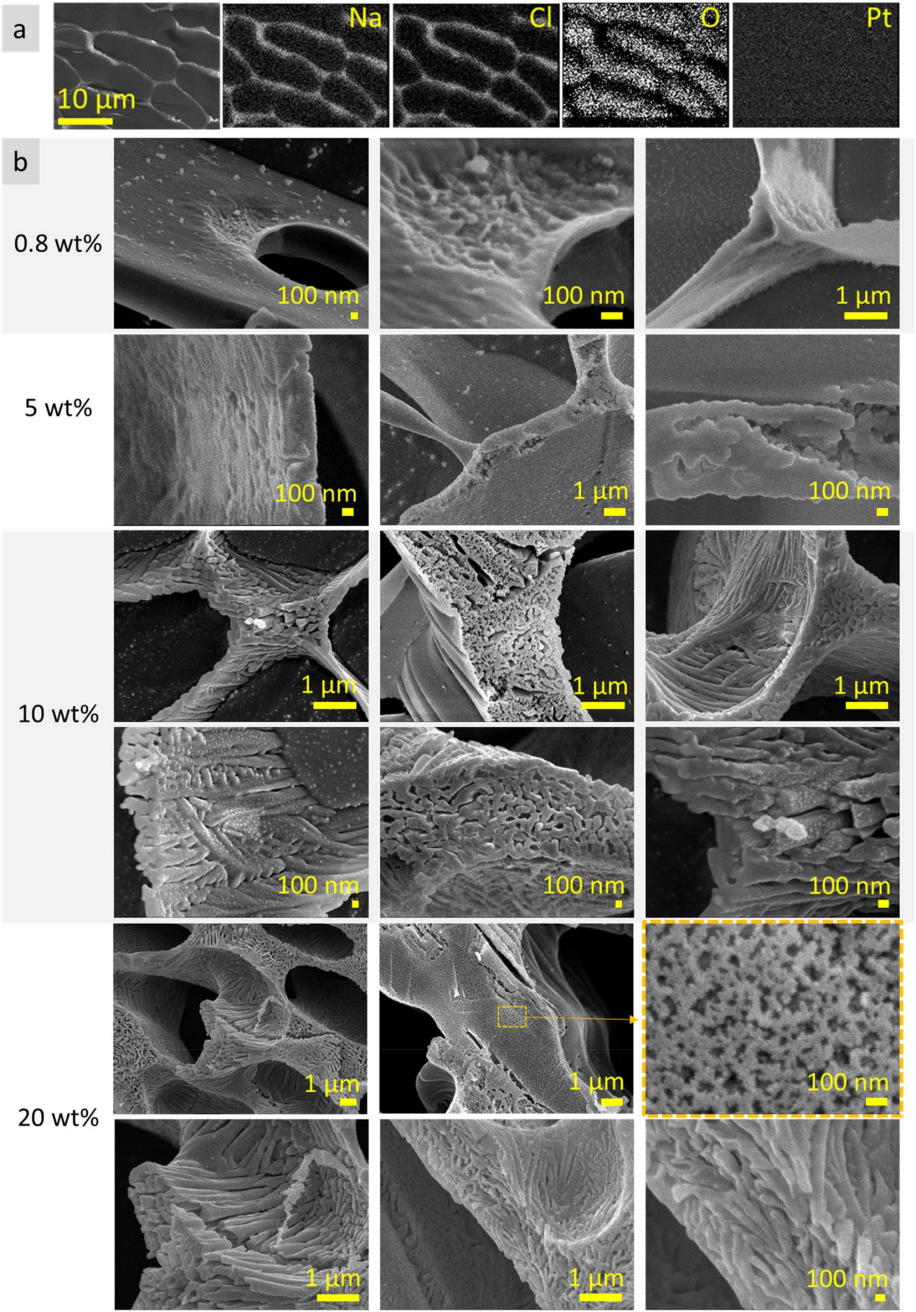
Cryo-SEM EDS elemental mapping of 10 wt% NaCl solution: Na, Cl, O and Pt elemental distribution. Pt was used for plasma sputtering, its homogenous distribution indicates that microstructural elements from the sample did not overtop surface influencing the electron flow (**a**). Cryo-SEM images of NaCl solutions (0.8–20 wt%): microstructure at nanoscale level (**b**).

Cryo-SEM elemental mapping indicated that Na and Cl elements were positioned predominantly in the microstructure walls, whereas O (from H_2_O)—between walls (Fig. 8a). Na and Cl elements are located predominantly in the microstructure walls (homogenous distribution of Pt element used for plasma sputtering indicated that microstructural elements did not overtop surface of the sample affecting the electron flow). Detailed investigation (at nanoscale level) of structure of these walls showed that they consist of roundish structures < 50 nm in size (Fig. 8b) (cryo-SEM EDS measurements “in point” for the observed spheres were not performed due to the technical limitation: at such a high magnification (× 100,000) electron beam energy melts and destroys analyzing area over the time (~ 30 s) needed to collect EDS spectrum). For the NaCl solutions in concentration range from 0.2 to 5.0 wt%, the roundish structures are randomly packed forming solvation walls, whereas at concentrations 10.0 or 20.0 wt% they form elongated wires (~ 100 nm thick, ≤ 3 µm length), which, in turn, are organized in complex pattern forming the walls of cell-like microstructure. Cryo-SEM images of NaCl solutions at microscale level demonstrate complex volumetric microstructure which, with increased concentration, transforms from discontinuous lamellar (at 0.2–1.5 wt%) to cell-like structure (at 2.0–20.0 wt%) (Fig. S5, S6). The three-dimensional (3D) microstructure of NaCl solutions may be described as a quantity of differently oriented domains of aggregated cell-like elements. The size of the domains is approximately 100–500 µm in each dimension. The thickness of the solvation walls increased with NaCl concentration. Detailed investigation of this feature is presented in Supplementary Fig. S7, Table S1-S3.

One more interesting feature noticed was deviation of Na and Cl atomic concentrations from stoichiometric ratio: amount of Na or Cl atoms prevailed depending of analyzed area. In some analyzed areas, the ratio between Na and Cl atoms was 1 : 1.88. Disproportion between Na and Cl atom distributions was observed on areas in the range 475–1230 µm^2^ (ca. 43.1 **∙** 10^−6^ mm^3^, X-ray penetration ~ 3.5 µm). Such inhomogeneity may cause a local predominance of certain reactions resulted in formation of acidic or basic compounds like HClO, NaOH, etc. This conclusion correlates with previous studies in which the pH microscale inhomogeneity of frozen NaCl solutions was confirmed^23,24^. In particular, the existence of pH gradient ∆pH = 0.2 within solvation walls in the frozen 20 mM NaCl solution was detected using ratiometric fluorescence microscopy^23^. Another study showed that freezing and thawing processes in the presence of NaCl could significantly change pH value causing decomposition of gallic acid^24^.

It can be assumed that observed highly-ordered microstructure of NaCl solutions reflects near-real state, i.e. the dynamic microstructure of neat water clusters and densely packed solvated Na^+^ and Cl^−^ ions is an inherent feature of this system. This supposition may be helpful in understanding of, e.g., electrolyte abnormalities.

## Discussion

It is generally accepted that water is a highly structured liquid due to an extensive network of hydrogen bonds; however, agreement does not exist on how the structure is to be defined^25,26^. In the past years, it was found that water molecules may form different types of structures, e.g., dimers, tetramers, hexamers and nanoclusters^27^. Such water structures have been visualized on 2D surfaces (on NaCl(001) films supported by a Au(111), NaCl(100) bilayer on Ag(111), Cu(110), surfaces, etc.)^27–31^. In particular, water was found to aggregate into various clusters with the dominant of water dimers on a Cd(0001) surface^27^. Interestingly, the amorphous ice nanoflakes were built exclusively from water dimers. It was demonstrated that the ultrahigh stability of water dimers results from the adsorption-induced enhancement of dipole moment of water^27^. In salt solutions, two types of water molecules coexist: non-interacting and directly interacting with ions forming dynamic hydration shell (solvate layers)^25,26^. The structure of water in solvate layers was shown to be different from that of non-interacting water revealing different properties (e.g., have a larger mean density than neat water)^26,32^. Hydrated ions may be regarded as rigid spheres on a picosecond timescale, so the solution can be imagined as bulk liquid water with suspended spheres^33^. Herein, changing in macroscopic properties of solutions (e.g., increase of viscosity) was usually related to altering of hydrogen-bond structure of neat water^34,35^. However, measurements of the orientational correlation time of water molecules in high concentrated Mg(ClO_4_)_2_, NaClO_4_, and Na_2_SO_4_ solutions by means of femtosecond pump-probe spectroscopy revealed that the addition of ions had no influence on the rotational dynamics of water molecules outside the first solvation shells of the ions^33^. The results obtained for pure water and highly concentrated solutions of NaClO_4_, Na_2_SO_4_ showed that the anisotropy decay of water-bonded OH groups was not affected by the presence of high concentrations of ClO_4_^−^, SO_4_^2−^, and/or Na^+^ ions. This indicated that, even in high concentrated salt solutions, ions with solvation shells coexists with pure bulk water, that correlates with our findings. Moreover, formation of ordering clusters of particles has been visualized along the flow direction in dispersions, and interpreted as the origin of shear thickening in colloidal dispersions^36^.

**Figure 9:**
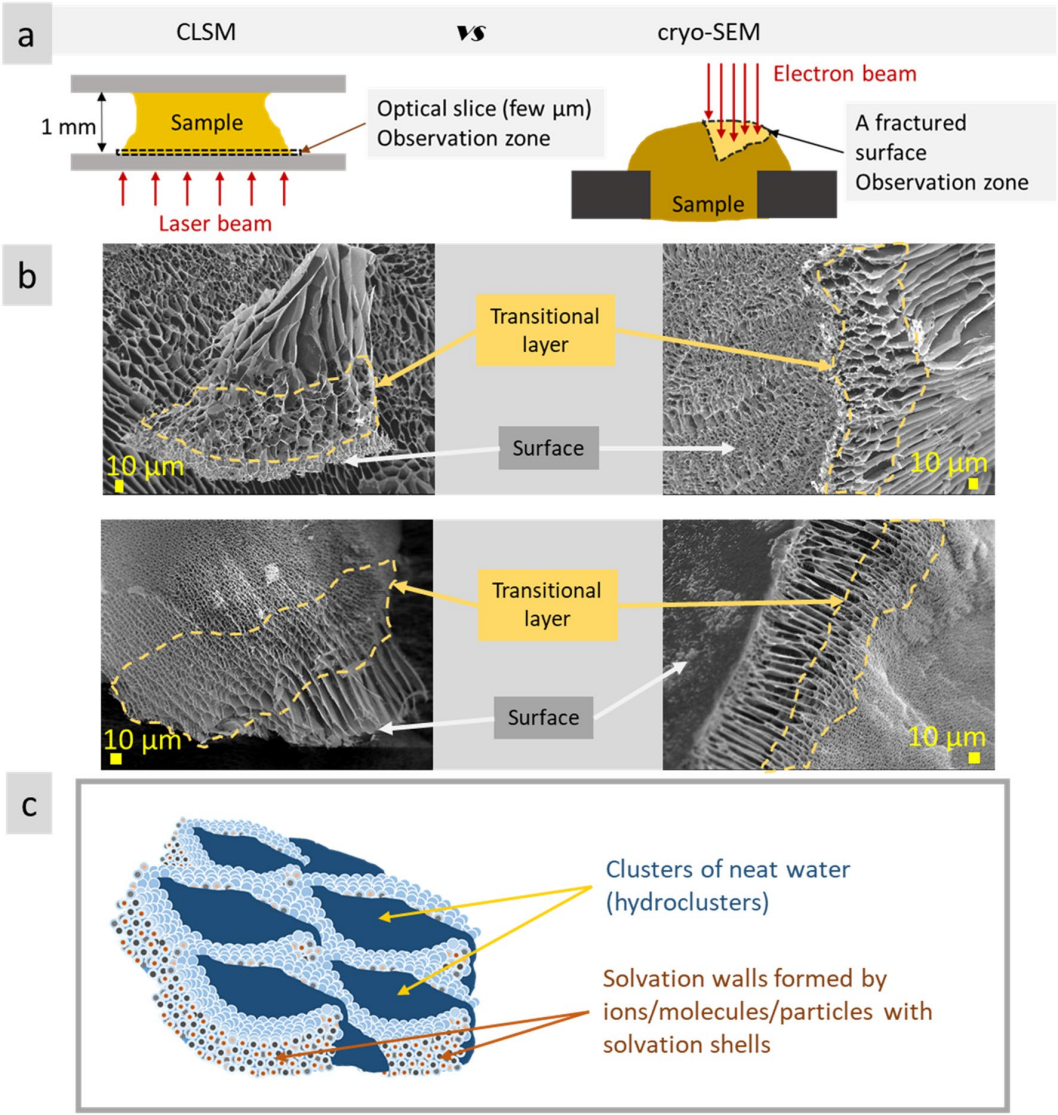
Scheme of sample observation at confocal LSM and cryo-SEM measurements (**a**); examples of microstructure scale transfer through transitional layers (circled by dotted line), from surface to bulk, in the polysaccharide gel at air/gel boundary^37^ (**b**); scheme of dynamic solvation microstructure (**c**).

In this study the microstructure of stable aqueous systems were investigated using cryo-SEM and confocal LSM techniques. The results showed concurrence of structural features observed by cryo-SEM and confocal LSM measurements for agar gel, mineral (montmorillonite and kaolin) clays, and NPs hydrocolloid. In particular, for agar gel such common microstructure features were volumetric structures with irregular shapes 20–400 µm in size, layered structures, highly ordered elongated and porous structures. For MAg NPs, a common feature was roundish shapes 20–100 µm in size. For kaolin clay, a characteristic feature was parallel alignment of kaolin micro plates, whereas for montmorillonite clay it was porous spongy microstructure. Besides similar features observed between the results of the two techniques used, there were also differences, such as inconsistency of microstructure general view and dimension. The latter may be prescribed to the different sample observation zones during the measurements: at cryo-SEM, bulk structure is observed (due to sample fracturing), whereas at confocal LSM study, a thin (few micrometers) layer near glass surface was investigated (e.g., at 1 AU depth is ~ 1 µm) (scheme in Fig. 9a). As known, bulk microstructure differ significantly from the surface layer. Figure 9b presents the examples of microstructure scale transition occurred in polysaccharide gel (air/gel boundary), from bulk to surface through intermediate layer. Scale transition may go from coarse-scale microstructure in the bulk to fine-scale on the surface and, *vise versa*, from the fine-scale in the bulk to coarse-scale on the surface (Fig. 9b)^37^. These observations indicate that the results of confocal LSM measurements of water-contained non-biological systems (i.e., without pronounced boundaries in water) strongly depend of glass/sample interaction (affinity between them, wettability, etc.). This conclusion is also supported by our results of confocal LSM measurements of montmorillonite clay with Atto 550 NHS described above. Montmorillonite clay with this fluorescence dye revealed highly parallel structures that was prescribed to improved affinity between clay and glass surface (for comparison, montmorillonite clay with fluorescein revealed only porous spongy structures). Thus, the microstructure of the near surface layer (substrate/sample, air/sample) differs significantly in scale and pattern from that in the bulk.

## Conclusions

Correlation between the cryo-SEM and confocal LSM measurements of stable water-contained systems (agar, kaolin, montmorillonite, MAg NPs) allowed to conclude that ordered microstructure is an inherent feature of these systems. It is supposed that NaCl solutions may also possess dynamic (femtosecond-level) ordered microstructure consisted of neat water clusters and densely packed solvated Na^+^ and Cl^−^ ions (proposed scheme in Fig. 9c). The supposition about microstructure existence may help in understanding of abnormal properties (e.g., density, viscosity, pH) of some aqueous systems (electrolytes, dispersions, etc.).

## Materials and Methods

### Materials

The analytical grade reagents of agar (CAS 9002-18-0, A1296), kaolin (CAS 1332-58-7), montmorillonite (CAS 1318-93-0), rhodamine B (CAS 81-88-9), fluorescein (free acid) (CAS 2321-07-5), CaCl_2_∙2H_2_O (CAS 10035-04-8), fluorescence dye Atto 550 NHS ester (MFCD05865403), dimethyl sulfoxide (DMSO) (CAS 67-68-5) were purchased from Sigma-Aldrich and used as received. Sodium chloride 99.9% pure (CAS 7647-14-5), ammonium hydroxide (CAS 1310-73-2) were purchased from POCH S.A. Ultrapure water (resistivity > 17 MΩcm) from a GZY-P10 water system was used throughout the experiments. For cryo-SEM measurements, deionized ultrapure sterile-filtered water (CAS 7732-8-5, Sigma-Aldrich) was used. Glass slides 24 × 50 mm (Menzel Gläser) and 18 × 18 mm (Zeiss), and rubber gasket (o-ring, 13 × 1 mm) were used for confocal LSM measurements.

### Preparation of solutions and dispersions

Hydrocolloid of ultrasmall iron oxide and silver (MAg) NPs which preparation, physicochemical and biological characterization have been provided^21^, was used in this study. MAg NPs hydrocolloids are able to form stable aqueous dispersions, and at high concentration (≥ 37 mg/ml), colloidal gels. In this study, fluorescent dye rhodamine B was added to the MAg hydrocolloid (hydrocolloid concentration: the NPs-38.2 mg/ml, rhodamine B-49.2 µg/ml) in order to improve contrast at confocal laser scanning microscopy (CLSM) imaging (to apply ultralow laser power that minimize sample heating and water evaporation during measurements). The concentration of rhodamine B in MAg hydrocolloid was 49.2 µg/ml. After addition of rhodamine B, MAg hydrocolloid was vortexed and stored in the dark at 4 °C until used.

To prepare agar gel (5 wt%) with rhodamine B, a rhodamine B solution (100 µl, 2.95 mg/ml) was mixed with 4900 µl of water, and combined with agar powder (250 mg). The concentration of rhodamine B in agar gel was 59 µg/ml. To solve agar, a termoresistant glass with prepared mixture was put into microwave oven for a few minutes avoiding boiling. Cooled rigid and transparent gel was stored in the dark place at 4 °C until used.

To prepare kaolin clay (27.5 wt%), 1.5 g of kaolin powder was mixed with 5000 µl of water, than 1.6 mg of fluorescein, 150 µl of 5 M NaOH, 300 µl of CaCl_2_ ∙2H_2_O (5 wt%) were added, mixed, sonicated for 2 h and left in the dark at room temperature for 48 h. The concentration of fluorescein in kaolin clay was 293.5 µg/ml. Prepared kaolin clay was stored in the dark at 4 °C until used.

To prepare montmorillonite clay (30 wt%), 1.5 g of montmorillonite powder was mixed with 4550 µl of water, than 1.6 mg of fluorescein, 150 µl of 5 M NaOH, 300 µl of 5 wt% CaCl_2_ ∙2H_2_O were added, mixed, sonicated for 2 h and left in the dark at room temperature for 48 h. The concentration of fluorescein in montmorillonite clay was 320 µg/ml. Prepared montmorillonite clay was stored in the dark at 4 °C until used.

To prepare montmorillonite clay with fluorescence dye Atto 550 NHS ester, 1 µl of dye stock solution (1 mg/100 µl DMSO) was diluted in 1 ml of water. Then, 200 µl of this solution was mixed with 300 µl of water, 150 mg of montmorillonite powder, 10 µl of 5 wt% CaCl_2_ ∙2H_2_O, 10 µl of 5 M NaOH. The concentration of Atto 550 NHS ester in montmorillonite clay was 3.85 µg/ml. Prepared montmorillonite clay was stored in the dark at 4 °C for 48 h before measurements.

Sodium chloride solutions (0.2–20 wt%) were prepared using ultrapure water.

### Cryo-SEM measurements

Cryo-SEM measurements were performed using scanning electron microscope JEOL 7001Fequipped with PP3000T Cryo System manufactured by Quorum Technologies (which includes SEM cold stage, anti‐contaminator, column mounted preparation chamber, PrepDek specimen preparation desk, turbo pumping stack and CHE3000 cold gas cooling system). The PP3000T Cryo System utilizes nitrogen slush plunge-freezing method for rapid sample freezing. Liquid nitrogen freezes at 63 K (− 210 °C) at wide range of pressure values. By plunging an object into slushed nitrogen more rapid cooling is obtained than plunging into liquid nitrogen at its boiling point (‒196 °C). High cooling speed prevents ice crystal growth fixing them on nucleation stage (severe nm in size).^2^ Following the protocol (PP3000T User Manual v.1.4), rapid specimen freezing was performed at the workstation (trademarked PrepDek), which includes slusher pots for specimen preparation and manipulation and the control electronics. Liquid samples were put as a drop (~ 30 µl), agar gel as a peace into a hole of specimen stub so that it protrudes above the surface, and mounted on shuttle. Following freezing, the specimen was transferred to the column mounted preparation chamber using cryo transfer device which enables it to be stored under vacuum keeping it clean and moisture free. The preparation chamber equipped with large cold surfaces provide the facilities to fracture, sublimate and plasma sputter coating of specimen. At the preparation chamber, the cold stage temperature was kept about ‒185 °C (at specimen fracturing and sputtering) with a stability of 1 °C, and chamber vacuum was in the range of 8.5·10^–4^ Pa. Sublimation (ice etching) was performed at ‒90 °C; duration varied depending on specimen and was chosen experimentally (typically between 5–50 min) unless specified. Plasma sputter coating was performed using Pt target. From the preparation chamber the specimen was subsequently transferred into the SEM chamber for observation. Inside the electron microscope is a cold stage that keeps samples frozen during observation. The temperature of the cold stage in the electron microscope was maintained at ‒190 ± 2 °C (vacuum level ~ 2.6·10^–5^ Pa). Secondary electron images of the frozen specimen were acquired at 5 keV. Energy-dispersive analysis (EDS) was performed by means of X-Max 80 mm^2^ Large Area SDD Silicon Drift Detector (Oxford Instruments) at 15 keV. Cryo-SEM measurement of NaCl solutions were performed using gold-sputtered specimen stub, duration of sublimation was 30–60 min at ‒ 90 °C; sublimation time for cryo-SEM EDS analysis was reduced to 10–15 min (to keep the specimen surface flat).

### Confocal LSM measurements

Imaging of agar gel, mineral clays and NPs hydrocolloid at room temperature (without freezing) was performed using confocal LSM Zeiss LSM 780 equipped with a femtosecond tunable infrared laser for two-photon excitation. The specimen (30 µl or 4 × 4 mm piece of gel) were placed on a glass slide (24 × 50 mm), with a rubber gasket (o-ring 13 × 1 mm) on top, inside the rubber gasket (to keep specimen volumetric during the measurement; thickness of a rubber pad ≈ thickness of specimen) and covered by another glass slide (18 × 18 mm) (to reduce water evaporation during measurements) (see Fig. 2a). The measurements of all the samples were performed using objective C-Apochromat 40x/1.20 W Corr M27 (numerical aperture 1.2) at low laser power (< 1% of nominal laser power; to reduce water evaporation and laser-induced changes in the specimen), gain 830. Line average mode was applied for image recording. The concentration of rhodamine B, fluorescein and Atto 550 NHS fluorescence dyes in the samples were mentioned above, in the section “Preparation of solutions and dispersions”. Agar gel with rhodamine B was measured at laser excitation wavelength 561 nm, and emission range 585–735 nm. MAg NPs hydrocolloid with rhodamine B was measured at excitation wavelength 514 nm, emission 536–674 nm, and reflection mode. The 2.5 dimensional (2.5D) images (axes *x*, *y*, intensity) were obtained using processing technique that allows to merge two images. Measurements of kaolin and montmorillonite clays with fluorescein were performed at excitation 405 nm, and emission was recorded in the range 416–493 nm. Montmorillonite clay with Atto 550 NHS ester was dropped between a glass slide and a cover slip (without rubber gasket), and measured at excitation wavelength 561 nm, emission 571–670 nm. All the images were recorded near the glass slide surface.

## Supplementary Information

Supplementary Information.

## Data Availability

All data generated and analysed during this study are included in this published article and its supplementary information files.
