# Supplementary material for: Cryo-SEM and confocal LSM studies of agar gel, nanoparticle hydrocolloid, mineral clays and saline solutions
Source: Sci Rep. 2022 Jun 15;12:9930. doi: 10.1038/s41598-022-14230-w (PMC9200766; doi:10.1038/s41598-022-14230-w)
Supplement: Supplementary file 1 — Supplementary Information. [file 41598_2022_14230_MOESM1_ESM.docx]

**Cryo-SEM and confocal LSM studies of agar gel, nanoparticle hydrocolloid, mineral clays and saline solutions**

Olena Ivashchenko^a^

*^a^ NanoBioMedical Centre, Adam Mickiewicz University, 61-614 Poznań, Poland*

Corresponding author: oleiva@amu.edu.pl

**SUPPLEMENTAL DATA**


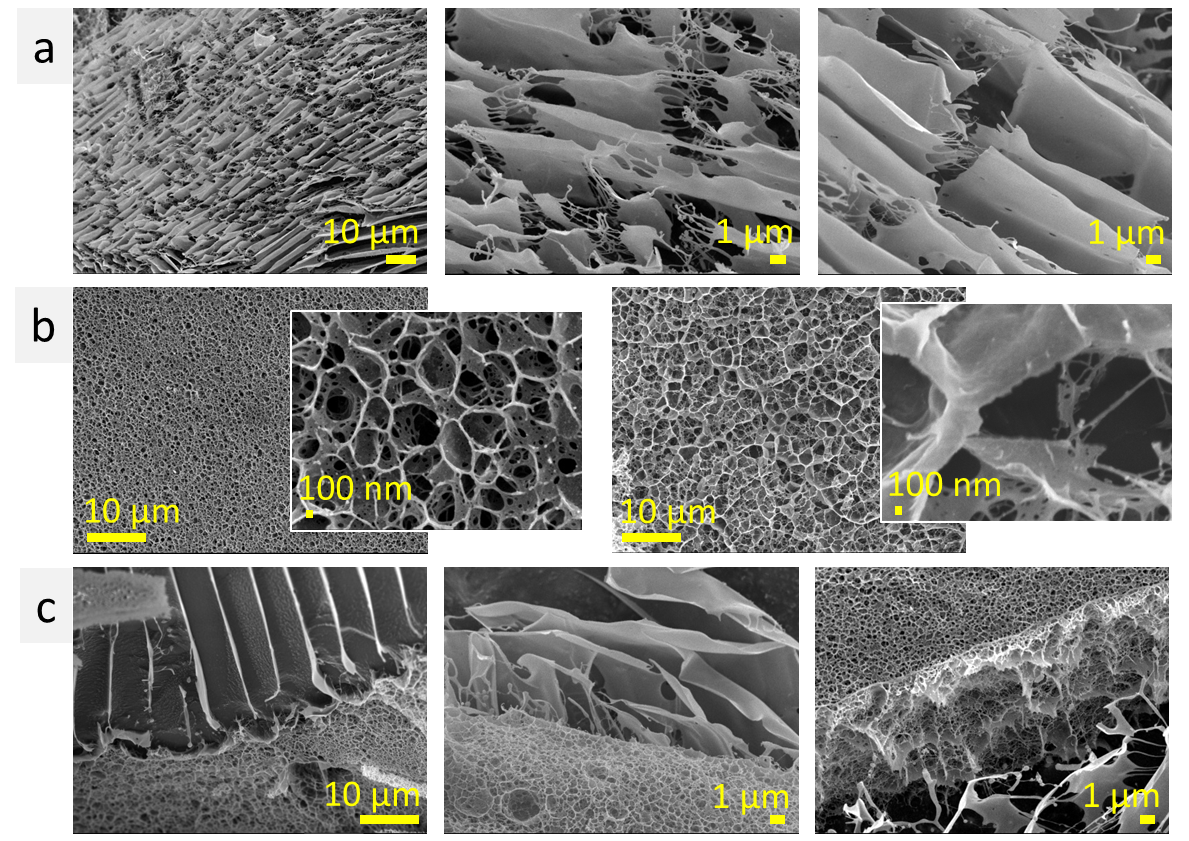


**Figure S1.** Cryo-SEM images of agar gel (5 wt%) with rhodamine: lamellae (a), porous (b) structures and borders between them (c).


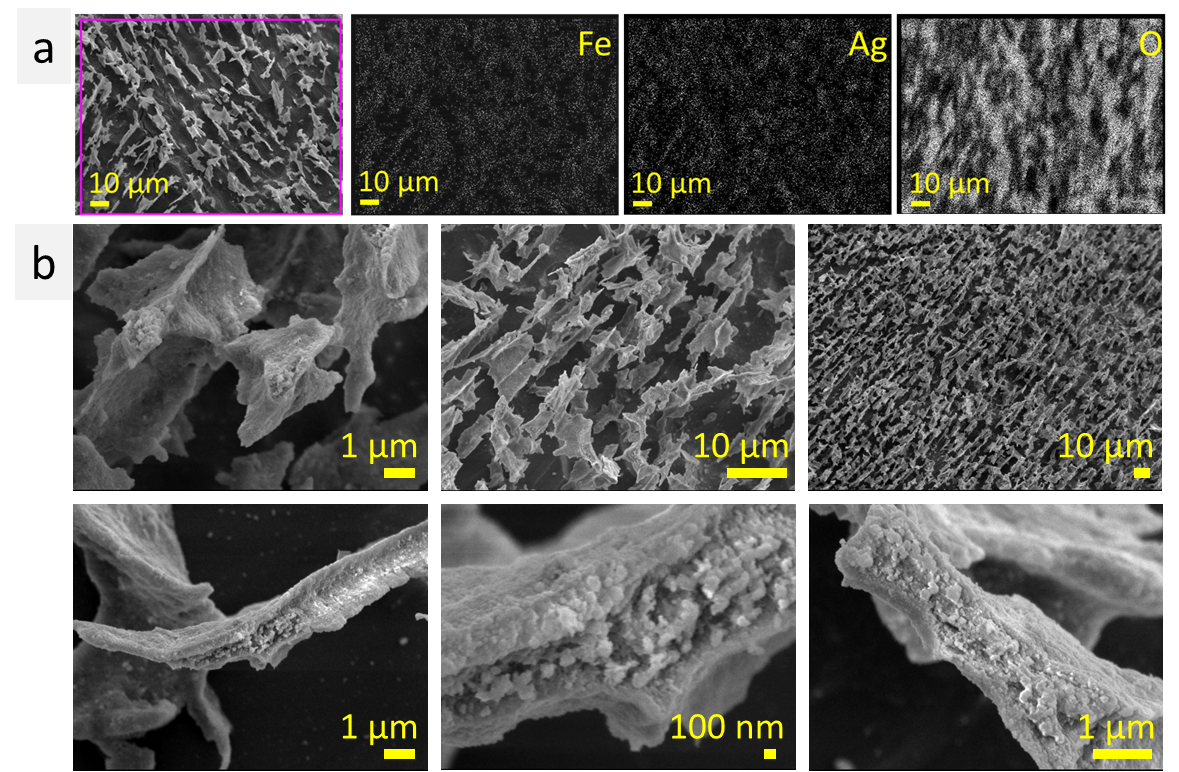


**Figure S2.** Cryo-SEM EDX mapping of MAg NPs hydrocolloid with rhodamine B: Fe, Ag and O elemental distribution (a); cryo-SEM images of MAg NPs hydrocolloid with rhodamine B (b).

**Figure S3.** Cryo-SEM images of ultrapure water (top row) and NaCl solutions in concentration range of 0.2 to 20.0 wt%.


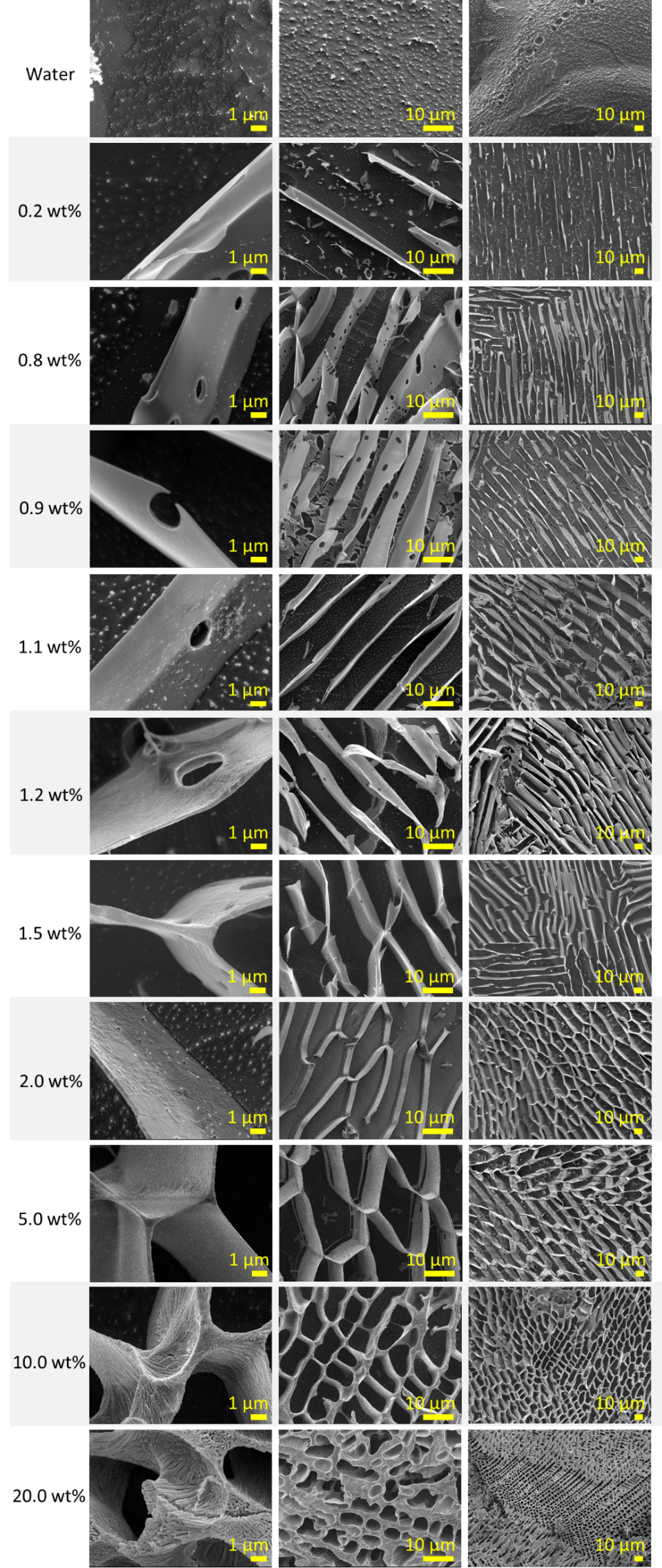


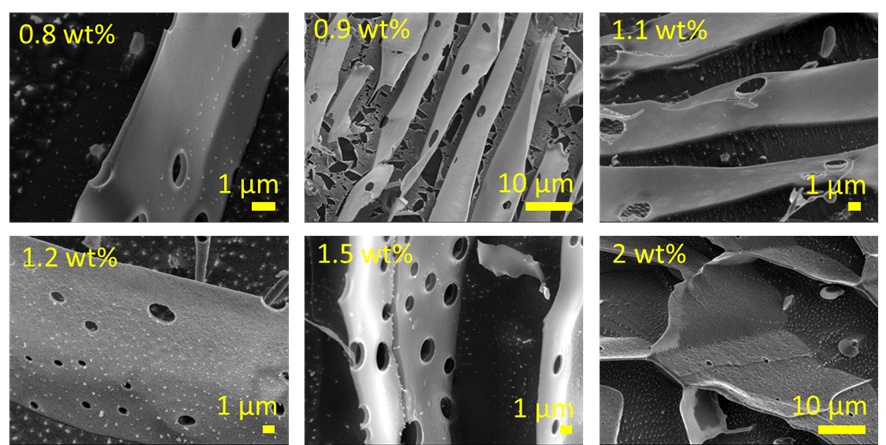


**Figure S4.** Microholes in the structure of solvate walls in 0.8-2.0 wt% NaCl solutions.


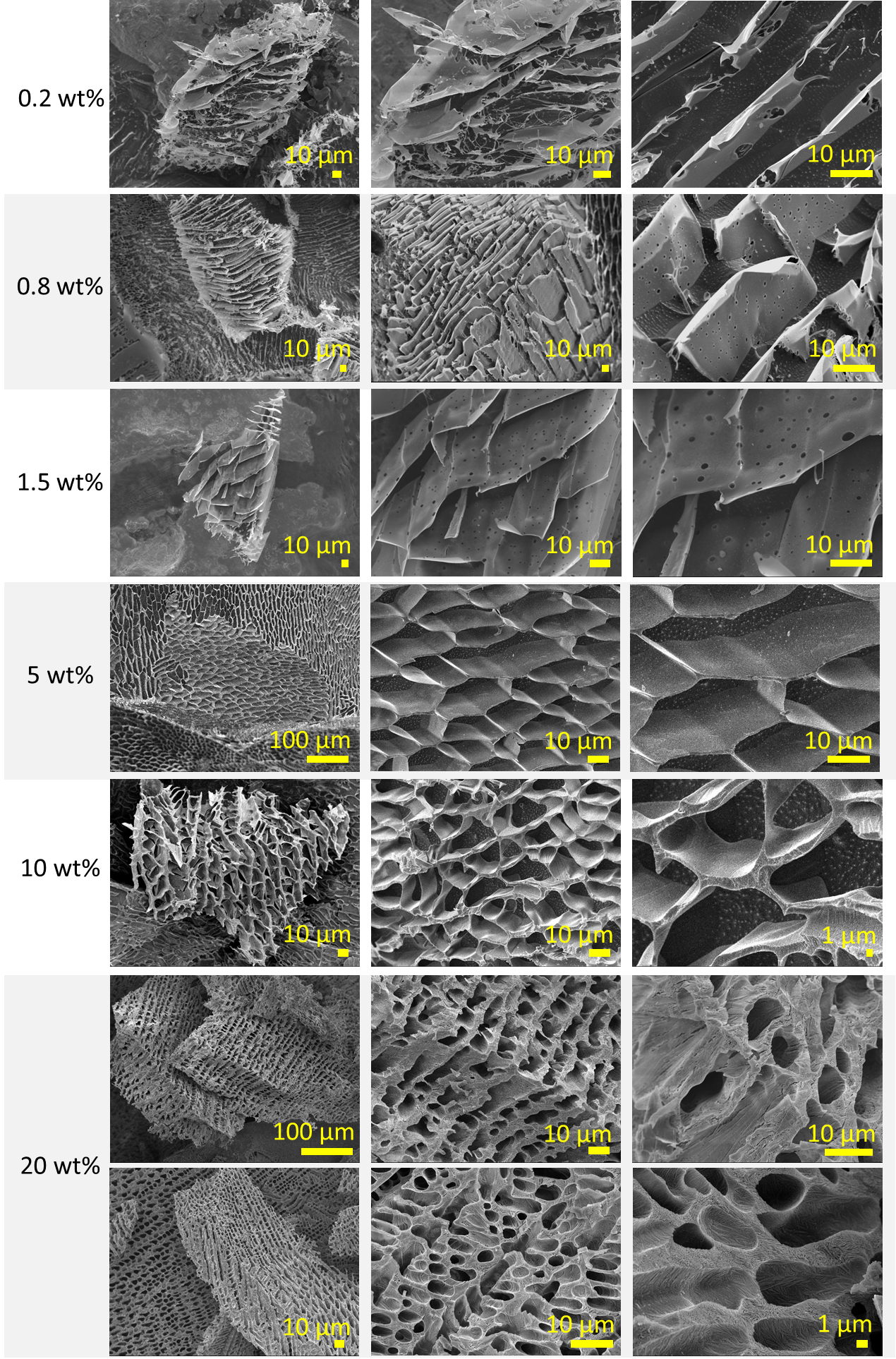


**Figure S5.** Cryo-SEM images of NaCl solutions (0.2-20 wt%): microscale level.

In order to check if the sublimation stage (performed at -90 °C) has an influence on microstructure formation, the cryo-SEM EDS elemental mapping was performed without sublimation stage: the sample was immersed into nitrogen flash (‒210 °C), fractured and transferred to SEM cryo stage for measurements (-173 °C, 15 kV). The Na and Cl elemental maps revealed the solvation microstructure patterns, whereas oxygen map showed that oxygen (from H_2_O) is distributed between solvation microstructure walls (Fig. S6). This result indicates that sublimation stage does not force microstructure formation.


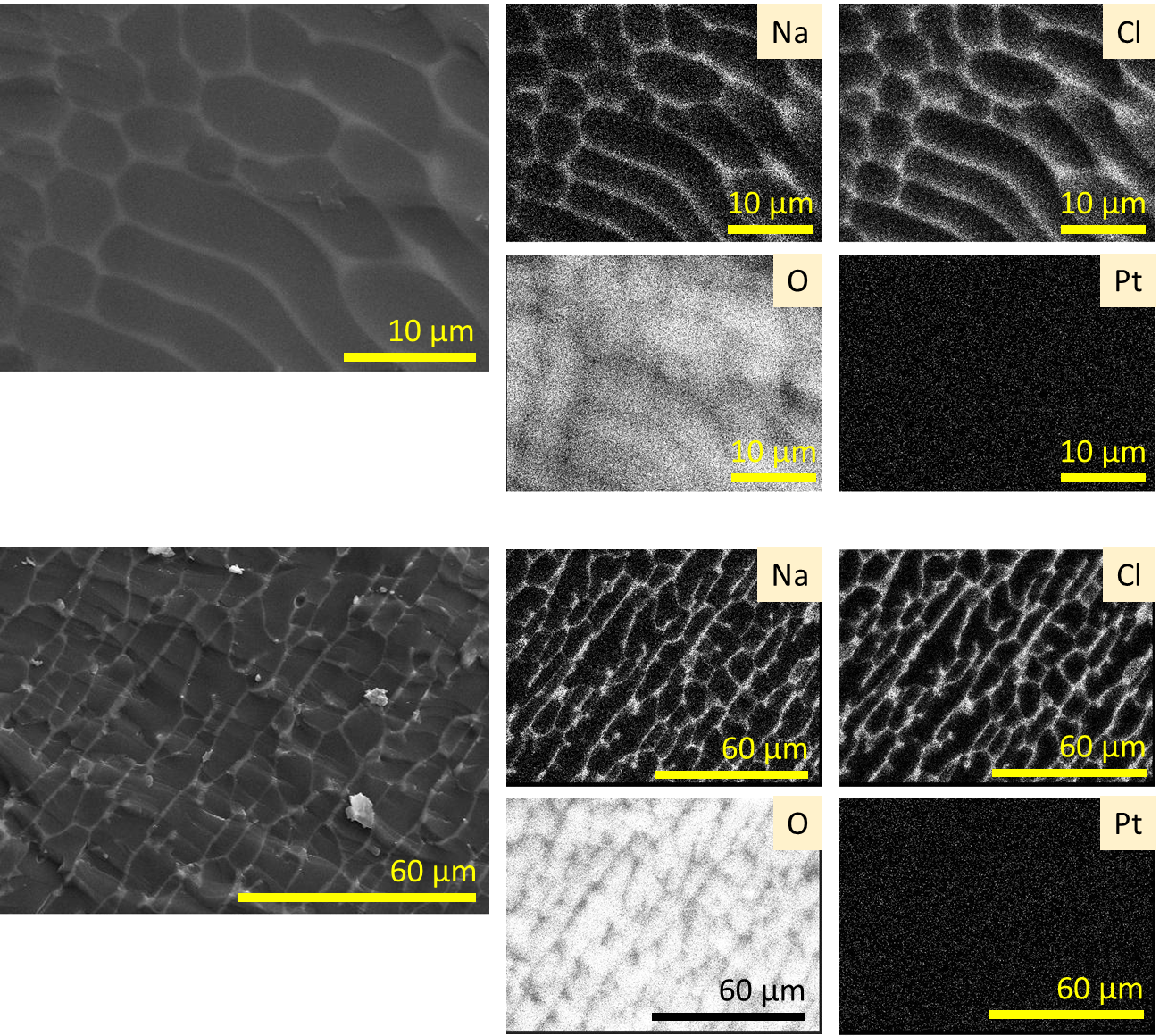


**Figure S6.** Cryo-SEM EDS elemental mapping of NaCl solution (10 wt%) plunged in nitrogen slush, fractured, and measured skipping sublimation stage. Pt element was used for sputtering; the maps of Pt element demonstrate it homogenous distribution on the surface.

Cryo-SEM EDS area analysis were performed for 10 wt% NaCl solution (Fig. S7, Supplemental Table S1-S3). Short sublimation time was used for this experiment (10-15 min, -90 °C), in order to avoid surface roughness that could affect the results. The measurements were performed on areas of 23×17 and 40×29 µm; the depth of beam penetration into the sample was estimated as ~2.5 µm (using Castaing’s formula). The analyzed areas were specified along solvation walls and in intermediate spaces (Supplemental Table S1-S3). The results indicated that Na and Cl atoms are located predominantly in solvate walls, whereas practically neat water (traces of Na and Cl detected) – in the space between these walls. Note, that the Na and Cl traces may be also originated from the solvate walls situated under the surface - they were not visible but detected by electron beam.


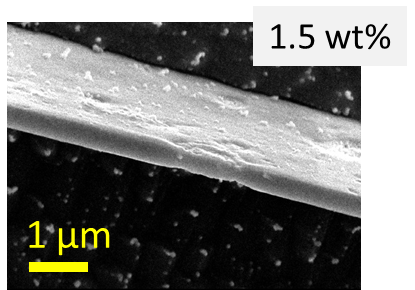


| NaCl concentration, wt% | Thickness, µm | | | | |
| --- | --- | --- | --- | --- | --- |
|  | Mean* | Min | Max | ±SD | Coefficient of variation |
| 0.2 | 0.057 | 0.01 | 0.10 | 0.029 | 0.52 |
| 0.8 | 0.118 | 0.04 | 0.42 | 0.072 | 0.61 |
| 0.9 | 0.151 | 0.06 | 0.29 | 0.058 | 0.38 |
| 1.0 | 0.108 | 0.05 | 0.22 | 0.061 | 0.56 |
| 1.1 | 0.193 | 0.08 | 0.40 | 0.071 | 0.37 |
| 1.2 | 0.352 | 0.11 | 0.90 | 0.231 | 0.66 |
| 1.5 | 0.293 | 0.03 | 1.02 | 0.181 | 0.62 |
| 2 | 0.292 | 0.06 | 1.18 | 0.169 | 0.58 |
| 5 | 0.393 | 0.08 | 1.58 | 0.262 | 0.67 |
| 10 | 0.786 | 0.01 | 4.06 | 0.718 | 0.91 |
| 20 | 1.955 | 0.28 | 5.07 | 1.071 | 0.86 |
| * n = 41-167 | | | | | |

**Figure S7.** Concentration dependent thickness of solvation walls in NaCl solutions, inset cryo-SEM image of solvate wall in 1.5 wt% NaCl solution, arrows demonstrate how thickness was measured (left); table presents descriptive statistics related to the measurements (right).

Table S1. Cryo-SEM EDS area analysis of 10 wt% NaCl solution; analyzed area 40×29 µm

| 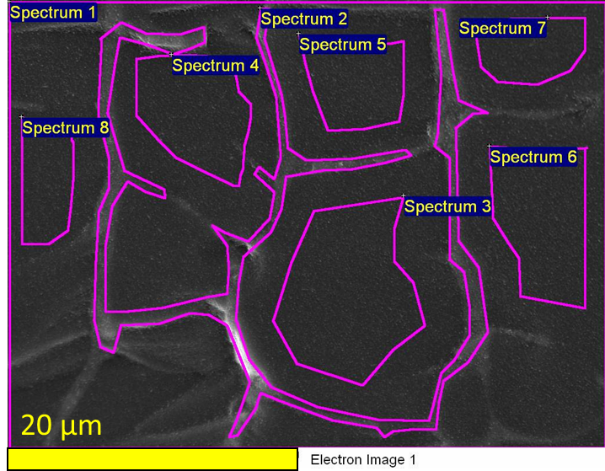 | Analyzed area | Mark on image | Element concentration, at% | | |
| --- | --- | --- | --- | --- | --- |
|  |  |  | O | Na | Cl |
|  | Solvate wall | Spectrum 2 | 88.66 | 3.94 | 7.40 |
|  | Intermediate space | Spectrum 3 | 100 | 0 | 0 |
|  |  | Spectrum 4 | 100 | 0 | 0 |
|  |  | Spectrum 5 | 100 | 0 | 0 |
|  |  | Spectrum 6 | 99.48 | 0 | 0.52 |
|  |  | Spectrum 7 | 99.47 | 0 | 0.53 |
|  |  | Spectrum 8 | 100 | 0 | 0 |

Table S2. Cryo-SEM EDS area analysis* of 10 wt% NaCl solution; analyzed area 23×17 µm

| 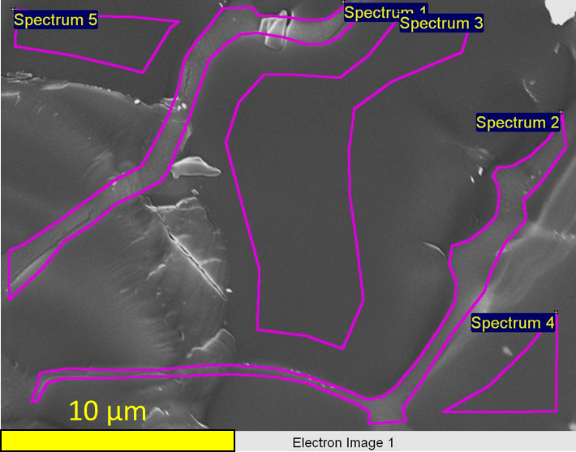 | Analyzed area | Mark on image | Element concentration, at% | | |
| --- | --- | --- | --- | --- | --- |
|  |  |  | O | Na | Cl |
|  | Wall | Spectrum 1 | 88.44 | 6.11 | 5.45 |
|  |  | Spectrum 2 | 88.31 | 6.16 | 5.53 |
|  | Intermediate space | Spectrum 3 | 99.74 | 0.26 | 0 |
|  |  | Spectrum 4 | 100 | 0 | 0 |
|  |  | Spectrum 5 | 99.75 | 0.17 | 0.07 |

* [It should be noted that cryo-SEM EDX area analysis provide the data not only from the surface, as may be concluded from the image, but also from under surface layer. Electron beam penetrates inside and detect backscattered electrons originated from an onion-like area under surface. According to Castaing's formula, penetration depth of the electron beam depends on accelerating voltage, sample density, atomic mass and number. Using the formula, analytical area of 10 wt% NaCl solution was estimated at approximately 2 µm. For cryo-SEM EDX measurements, sublimation time was reduced to 10-15 min in order to obtain sample with “flat” surface, without protruding parts. This allowed to avoid disturbances caused by them (e.g., insufficient electron dead time).]

Table S3. Cryo-SEM EDS area analysis of 10 wt% NaCl solution, analyzed area 23×17 µm

| 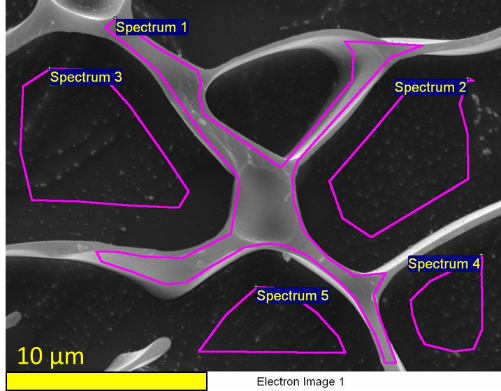 | Analyzed area | Mark on image | Element concentration, at% | | |
| --- | --- | --- | --- | --- | --- |
|  |  |  | O | Na | Cl |
|  | Wall | Spectrum 1 | 81.15 | 10.49 | 8.36 |
|  | Intermediate space | Spectrum 2 | 98.99 | 0.63 | 0.38 |
|  |  | Spectrum 3 | 98.73 | 0.77 | 0.50 |
|  |  | Spectrum 4 | 98.85 | 0.76 | 0.38 |
|  |  | Spectrum 5 | 98.11 | 1.08 | 0.81 |
